# Genetics of breast cancer in African populations: a literature review

**DOI:** 10.1017/gheg.2018.8

**Published:** 2018-05-11

**Authors:** A. Abbad, H. Baba, H. Dehbi, M. Elmessaoudi-Idrissi, Z. Elyazghi, O. Abidi, F. Radouani

**Affiliations:** 1 Laboratoire d’écologie et d'environnement- Faculté des Sciences Ben M'sik, Université Hassan II de Casablanca, Casablanca, Morocco; 2 Virologie Médicale et Laboratoire de BSL-3, Institut Pasteur du Maroc, Casablanca, Morocco; 3 Laboratoire de Biologie et santé – URAC34, Modélisation Moléculaire et Contrôle Qualité, Faculté des Sciences Ben M'sik, Université Hassan II de Casablanca, Morocco; 4 Laboratoire d'Immuno-Virologie, Institut Pasteur du Maroc, Casablanca, Morocco; 5 Laboratoire de Pathologies Cellulaires et Moléculaires- Faculté de Médecine et de Pharmacie de Casablanca, Université Hassan II de Casablanca, Morocco; 6 Laboratoire de Génétique Médicale, CHU IBN ROCHD, Casablanca, Morocco; 7 Laboratoire des Hépatites Virales, Unité de Virologie, Institut Pasteur du Maroc, Casablanca, Morocco; 8 Physiology and Physiopathology Team, Immunogenomic and Bioinformatic Unit, Faculty of Sciences, Genomic Center of Human Pathologies, Mohammed V University of Rabat, Morocco; 9 Chlamydiae and Mycoplasma Laboratory, Institut Pasteur du Maroc, Casablanca, Morocco; 10 Human Molecular Genetics and Medical Genomics Laboratory, Institut Supérieur des Professions Infirmières et Techniques de Santé (ISPITS) de Casablanca, Ministère de la Santé, Morocco

**Keywords:** Africa, breast cancer, genetics, genetic variability

## Abstract

Breast cancer (BC) is one of the most complex, diverse and leading cause of death in women worldwide. The present investigation aims to explore genes panel associated with BC in different African regions, and compare them to those studied worldwide.

We extracted relevant information from 43 studies performed in Africa using the following criteria: case-control study, association between genetic variations and BC risk. Data were provided on mutations and polymorphisms associated with BC without fixing a specific date. Case-only studies and clinical trials were excluded.

Our study revealed that the majority of African BC genetic studies remain restricted to the investigation of BRCA1 and BRCA2 genes and differences in their mutations spectrum. Therefore, it is necessary to encourage African researchers to characterize more genes involved in BC using methods generating global information such as next-generation sequencing in order to guide specific and more effective therapeutic strategies for the African community.

## Introduction and background research

Breast cancer (BC) is one of the most complex and diverse diseases, it represents the leading cause of death in women worldwide among other cancers and infectious diseases [1]. Along with ovarian cancer, they constitute the most common forms of cancer in the developed and developing countries [2].

Since BC is considered as a public health problem in most countries as stated by the World Health Organization (WHO) [3]. Several studies have been conducted, either to establish the link between this malignant tumour and its host, also to understand its genesis and its response to treatments and drug effects [4]. According to the 2014 World Health Cancer report, they estimated 14 million new cases and 8.2 million deaths due to cancer worldwide [5]. Currently, 690 000 new cases are being diagnosed annually in the developed regions with around 92 000 new cases in Africa [6]. Even though the incidence for BC is high, the rate of mortality has been decreasing over the past 25 years [7]. The last update of the global burden of cancer – International Agency for Research on Cancer (GLOBOCAN-IARC) [8] also reported that the prevalence of BC in adult populations shows a high rate in Asia (36.7%), followed by Europe (29.1%), North America (17.2%), Africa (7.0%) and Latin America (8.8%). The incidence of mortality has been likely the same in Asia (39%), Europe (27.5%), North America (15.3%), Latin America (9.1%) and Africa (8%). By 2020, the forecast of BC will continue its increase slowly in the five continents from 1 651 872 in 2012 to 1 956 124 cases alongside with number of mortality going from 517 578 cases in 2012 to 617 479 [8]. The growing prevalence of BC, especially in Africa, incriminates many factors, including hormonal, toxic or genetic factors. In this context, many genes such as *BRCA1/2,TP53, HER2* and *CHEK2* have been studied to characterize mutations specific to each population.

The BC screening by mammography can reduce the cancer-specific mortality. The value of other screening methods such as the genetic detection of familial and non-familial mutations is relevant and explain a large part of BC heredity. Although, the lack of observations of such gene mutations, particularly in Africa, is one of the reasons for the growing disparity between the sensitivity of diagnosis and rate of variation. Besides genetic variation associated with inheritance of some genes with defined penetrance of BC [4], the determination of other risk factors leading to the genetic variability of the disease is an area that needs to be considered for accurate detection of BC in Africa.

Epidemiological studies have provided great support to understand the genetic variability and risk factors among populations around the world to facilitate the accuracy of diagnosis, medical support and drug response. Therefore, the aim of this work is to provide a panel of genes associated with BC in the African populations. This panel of genes will be extracted through a literature review. Our investigation could be useful both for future epidemiological studies and enable decisions on new research and management strategies for BC genetic studies.

## Methods

We searched in the databases PubMed, Scopus and Web of Science for studies reporting genetic variations implicated in BC in Africa. The search was conducted based on specific Medical Subject Headings (MeSH) and keywords, which are BC, genetics, gene, mutation, association and Africa. All languages were searched initially, but only English language studies were selected.

**Fig. 1.**
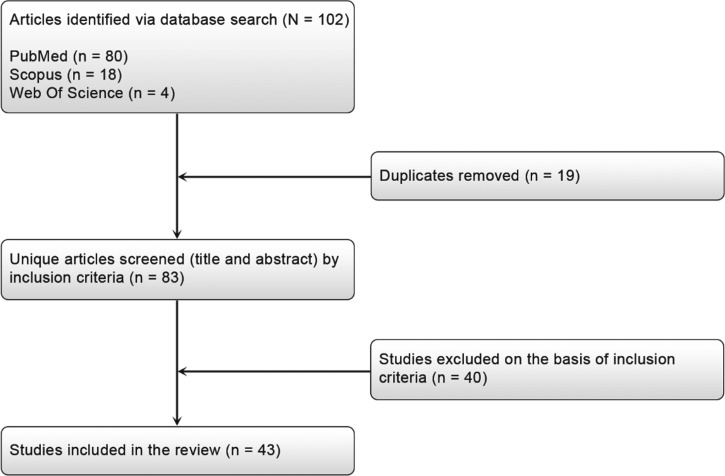
Flow diagram for identifying studies for assessment of breast cancer mutations in Africa.

We extracted relevant information from each study using a standardized form and we included studies in this review only if the following criteria were met: a case-control study on the association between a variation (SNP, InDel, CNV…) and BC risk, and data were provided on mutations and polymorphisms associated with BC. We excluded case-only studies and clinical trials. The study selection process and flow diagram for identifying studies is detailed in Fig. 1.

In order to extract most of studies regarding BC and genetics, we identified published articles without fixing a specific date. The strategy adopted was applied for African populations to evaluate African BC specificities. We also searched the data concerning other continents, this served to compare and discuss African results.

## Results

The African continent is commonly divided by the United Nations (UN) into five regions or sub-regions: Northern, Western, Central, Eastern and Southern Africa (http://www.un.org/esa/population/publications/worldageing19502050/pdf/96annexii.pdf). Therefore, we used this division to present the results retrieved from different databases.

### Northern Africa region (NA)

For this region, we summarized studies on BC mutations in Algeria, Tunisia, Egypt and Morocco. Only Libya was excluded for lacking relevant genetic studies on BC. In total, 28 major studies have been conducted in NA region, including more than 3700 BC cases and showing that *BRCA1/2* genes were the most investigated for their association with BC. Other genes such as *HER2, P53, APOBEC3* and *MTHFR* were also studied.

In Algeria, three major studies have investigated the carried mutations on *BRCA1* and *BRCA2* genes in both sporadic and familial cases. Uhrhammer *et al.* [9], using complete gene sequencing found one BC founder mutation in *BRCA1* gene (c.798_799delTT) in Algerian population with 9.8% familial cases and 36.4% sporadic cases. This mutation has been reported as the first non-Jewish founder mutation to be described in NA.

In another study performed by Cherbal *et al.* [10], over 101 individuals from 79 breast and ovarian cancer families were examined for unclassified variants (UVs) and polymorphisms in the BRCA1 and BRCA2 genes by Single-strand conformation polymorphism (SSCP) or High-Resolution Melting (HRM) curve analysis, followed by direct sequencing. The result revealed 168 UVs and polymorphisms in *BRCA1/2* genes, 68 in *BRCA1* and 100 in *BRCA2*. Cherbal and colleagues also performed *BRCA1/2* mutation screening by HRM curve analysis and direct sequencing in 86 individuals from 70 Algerian BC families with history of BC, five mutations were detected in BRCA1 (c.83_84delTG, c.181T>G, c.798_799delTT, delEx2, delEx8) and 57 UVs/SNPs were revealed in both BRCA1 and *BRCA2* [11]. In a recent study, Henouda *et al.* [12] found five mutations in 40 different Algerian families with early BC. Among them, five mutations were identified in *BRCA1* (c.1817del, Del exon 2, c.4065_4068del, c.5332 + 1G>A, c.5117G>C) and nine in *BRCA2* (c.7654dupA, c.1528G>T, Del exons 19-20, c.6450del, c.7462A>G, c.1504A>C, c.5117G>C, c.5939C>T, c.1627C>A).

In Tunisia, a *BRCA1* study on nine Tunisian patients with hereditary BC was carried out in order to evaluate the implication of the *BRCA1* and DNA mitochondrial mutations and revealed that the mitochondrial mutation 315.insC was strongly implicated in two unrelated patients [13]. Mahfoudh *et al.* [14] performed a screening for germline mutations of *BRCA1* in 16 Tunisian high-risk BC families, where six families were found with *BRCA1* mutations: three truncating mutations were described in *BRCA1* (c.798_799delTT, c.3331_3334delCAAG, c.5266dupC), one specific to Tunisian population (c.212 + 2insG) and the c.798_799delTT was suggested to be a Tunisian founder mutation based on its frequency (18%). Mestiri *et al.* [15] screened 12 Tunisian women with familial or sporadic BC for *BRCA1* gene mutations and the 1294del40 mutation of *BRCA1* was found only in a patient with non-familial BC. In contrast, the 185delAG mutation was absent in all cases of BC. Troudi *et al.* [16] studied the *BRCA1/2* genes in 36 Tunisian patients with breast and/or ovarian cancer. The results revealed 90% of cases with deleterious mutations. In *BRCA1* four mutations (c.211dupA, c.4041delAG, c.2551delG and c.5266dupC) were detected in two unrelated patients, the c.5266dupC mutation was described for the first time in a non-Jewish Ashkenazi population. In addition, two frame-shift mutations (c.1309del4 and c.5682insA) were observed in *BRCA2*. Furthermore, Riahi *et al.* [17] performed a screening on *BRCA1/BRCA2* genes in 48 patients by direct sequencing, the result revealed 12 mutations including three in *BRCA1* (c.211dupA, c.5266dupC and c.1504_1508delTTAAA) and two novel mutations in *BRCA2* (c.1313dupT and c.7654dupT). In a recent work, the same group proposed a cumulative mutation analysis with data from three Tunisian studies including 92 Tunisian women, the results revealed two recurrent mutations (c.211dupA and c.5266dupC) in 76% of BRCA1-related families and three recurrent mutations (c.1310_1313del, c.1542_1547delAAGA and c.7887_7888insA) in 90% of BRCA2-related families [18]. Hadiji-Abbes *et al.* [19] identified a novel in-frame deletion (5456del6 bp) in the *BRCA2* gene in an early onset woman with BC without family history.

**Fig. 2.**
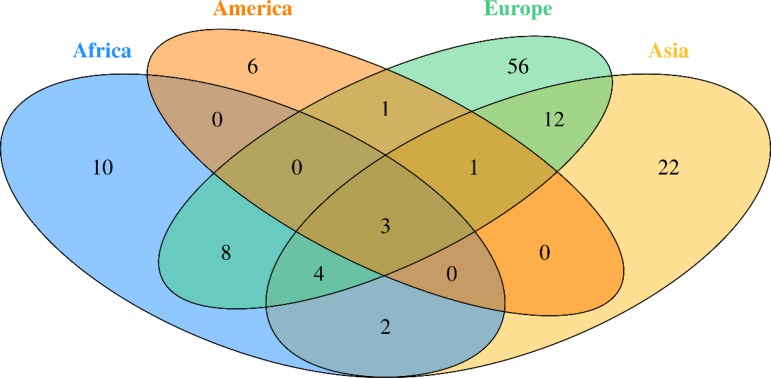
Venn diagram of studied Breast Cancer genes in different continent

*HER2* gene has been reported as major factor in BC development and progression [20]. Therefore, Kallel *et al.* [20] focused on this gene by analysing three polymorphisms in 148 cases and 290 controls from the Tunisian population. The Ile(655)Val mutation was found to be significant in 90% of cases. The noncoding SNP (rs903506) and the H(AC)I4 mutation were also implicated. The association of Ile(655)Val with BC has been confirmed by a further meta-analysis from 27 case-control studies [21].

A study concerning the *P53* genewas conducted in order to investigate the association of *p53* Arg72Pro, Ins16 bp and G13964C polymorphisms and their haplotypes with BC risk in 159 Tunisian female patients. The results revealed that these mutations were associated with familial BC risk in this population [22].

The *ESR1* and *ESR2* genes were also studied by Kallel *et al.* [23] in 148 Tunisian BC patients and 303 controls using the polymerase chain reaction-restriction fragment length polymorphism (PCR-RFLP) method. The *ESR1* 2014 ***G*** allele showed significant association with BC risk (*p*  = 0.025).

In Egypt, Bensam *et al.* [24] screened *BRCA1* and *BRCA2* in 20 Egyptian patients with BC. Mutations were detected in 44% of the studied population, with 18% in the *BRCA1* gene (185AGdel, 624C>T) and 26% attributed to *BRCA2* (999TCAAAdel, 2256T>C, 8934G>A).

The *P53* gene has also been studied in the Egyptian population by El-Ghannam *et al.* [25], their study focused on mutation detection in 30 patients with BC using flow-cytometry, PCR-SSCP and sequencing. *P53* mutations including A218 T, R279G, S297X and Y159X were detected in 17% of patients.

In another study, Hussein *et al.* [26] examined the relation between the *PON* L55 M and Q192R polymorphisms with BC risk in Egyptian females and analyzed their relation to clinic-pathological parameters of BC. Both SNPs were shown to be significantly associated with an increased risk of BC. However, when they conducted a study in order to evaluate the association of two polymorphisms in *XPD* (Asp312Asn) and *XRCC1* (A399G) on 100 BC Egyptian females, they did not detect an association between the *XRCC1* gene mutation and BC [27].

In Morocco, three studies targeted BRCA1/2 mutations, the first one was conducted by Laarabi *et al.* [28], and was interested in analyzing BRCA1/2mutations in five healthy women belonging to three families with an elevated risk of BC, this investigation revealed that three asymptomatic women were found to be carriers of BRCA1/2 mutations. The second study was performed by Tazzite *et al.* [29] on a Moroccan cohort of 40 women, diagnosed with BC and a familial history of breast/ovarian cancer, or aged less than 40 years old showed that 25.64% of patients carried *BRCA1/2* mutations. The third study, conducted by Laraqui *et al.* [30] on 121 Moroccan women diagnosed with BC, only *BRCA1* status was investigated and revealed that mutations were found in 36.1% of familial cases and 1% (1/102) of early-onset sporadic. The result of these three studies showed 14 BRCA1/2 point mutations; nine in *BRCA1* (c.68_69delAG, c.181T>G, c.798_799delTT, c.3279delC, c.2805delA, c.1016dupA, c.4942A>T, c.5062-5064delGTT, c.5095C>T) and five in *BRCA2* (c.517-1G>A, c.3381delT, c.5073dupA, c.7110delA, c.7235insG).

Considering the potential function of driver and *APOBEC3* genes in the process of tumorigenesis in BC, it is possible that germline variations and copy number variations (CNV) in those genes could influence the risk of BC. In this term, a case-control study was conducted in a group of Moroccan women targeting 36 SNPs in 13 genes (*APOBEC3A, APOBEC3B, ARID1B, ATR, MAP3K1, MLL2, MLL3, NCOR1, RUNX1, SF3B1, SMAD4, TBX3, TTN*). The analysis showed that 12 SNPs in eight driver genes, four SNPs in *APOBEC3B* and one SNP in *APOBEC3A* were associated with BC risk and/or clinical outcome at the significance level of 0.05. RUNX1_rs8130963 (*p*  =  0.0005), TBX3_rs8853 (*p*  =  0.0003), TBX3_rs1061651 (*p* =  0.0002), TTN_rs12465459 (*p* =  0.0009), were the most significantly associated SNPs with BC risk. A strong association with clinical outcome was detected for SMAD4_rs3819122 with tumour size (*p* =  0.009) [31].

Another study was conducted to investigate if *MTHFR* C677T polymorphism modulates the risk of developing BC in Moroccan women. Results showed a positive correlation between the *MTHFR* C677T polymorphism and progesterone receptor expression (*p* =  0.04). There was a significant association between C677T polymorphism and BC risk in both additive (*p* =  0.007) and dominant (*p* =  0.008) models [32].

Many studies were also conducted to investigate the implication of three genes described as a high penetrance BC susceptibility. The *PIN3* Ins16 bp polymorphism of the *TP53* gene [33], the *ABCB1* C3435T polymorphism [32] and the +936C/T VEGF-A polymorphism [34]. However, there was no evidence of a significant association between these polymorphisms and risk of BC.

### Southern Africa (SA) region

No founder genes have been described in BC patients of the four countries (Botswana, Lesotho, Namibia and Swaziland) included in SA's region. Whereas, the studies performed among South Africa's BC patients revealed variants in the two major genes *BRCA1*&*BRCA2* and in five other genes deemed intermediate (*CHEK2*, *PALB2*) and minor (*RAD50, MTHFR, hMLH*).

Reeves *et al.* [35] were the first to report the role of *BRCA1* in 90 SA BC families in 2004. Thereafter, Francies *et al.* [36] studied 108 sporadic patients with 78 women premenopausal and 30 triple negative breast cancer (TNBC) from the four South African ethnic groups, to determine the mutations frequency in *BRCA1, BRCA2* and *PALB2* and evaluate the presence of the *CHEK2*. This study used the next-generation sequencing (NGS) approach in combination with Multiplex Ligation-dependent Probe Amplification (MLPA) to detect large rearrangements in *BRCA1* and *BRCA2*. The result revealed the BRCA2 (c.7934delG) Afrikaner founder mutation, the *BRCA2* variant (c.9875C>T) and the *CHEK2* mutation (c.1100delC). *PALB2* variants (c.118A>G, c.2845T>C) were also described as probably damaging [36]. The two studies mentioned previously and carried out on the *BRCA1* gene reported nine following mutations in a South African population: c.181T>G, c.212G>A, c.3593T>A, c.1155G>A, c.1953_1954insA, c.1843_1845delTCT, 1493delC, 185delAG, 4957insC, 5382insC, E881X and S451X [35, 36].

Sluiter *et al.* [37] also identified *PALB2* as a BC susceptibility gene, and the related mutations double the BC risk with moderate to low penetrance. In a cohort of 48 young South African BC patients unselected for family history of BC, the authors determined the involvement of *PALB2* mutations and they identified a novel truncating mutation, c.697delG (V233fs).

*MTHFR, RAD50* and *hMLH1* genes were also showed to be involved in the development of BC in this region of Africa [38, 39].

### Western Africa region

In this region of Africa, few studies focused on the genetic risk factors for BC. These studies were performed only in Senegal and Nigeria, and were restricted on the description of the mutations in *BRCA1, BRCA2* and *LEPR* genes.

In Nigeria, genetic studies revealed that *BRCA1* and *BRCA2* are the most prevalent cause of BC. The overall *BRCA1/2* mutation rate is 11%, which is the highest reported rate for any BC cohort from a non-founder population unselected for family history, ethnicity or age of onset in Nigeria. Unexpectedly, only 7.1% of patients carried *BRCA1* mutations and 3.9% individuals were *BRCA2* mutation carriers. A total of 48 mutations were found in *BRCA1/2* (31 in *BRCA1* and 17 in *BRCA2)*, including non-sense, missense, frame-shift and splice-site mutations. Deleterious mutations like Y101X, 1742insG, C64Y, 4241delTG and del Ex 21 were the most commonly carried BC-related mutations in exons 11, 12 and 21 of the *BRCA1* gene. The *BRCA2* gene in the Nigerian population carried the 1538delAAGA, 2630del11 and 9045delGAAA mutations in exons 10, 11 and 22, respectively [40].

The *LEPR* Gln223Arg polymorphism was also investigated in premenopausal Nigerian women carrying at least one *LEPR* 223Arg allele. This investigation revealed that the heterozygote Gln223Arg and mutant homozygote Arg223Arg have no association with postmenopausal BC risk (*p* = 0.68) [41]. Premenopausal Nigerian women carrying at least one *LEPR* 223Arg allele were at a modestly increased risk of BC (*p* = 0.07) [41].

In Senegal, BC studies revealed that the *BRCA1* gene is the most common genetic risk factor for the disease development in Senegalese women. Indeed, a novel deleterious mutation (c.1949_1950delTA) has been described [42].

### Eastern Africa region

In this region, our bibliographic research revealed that only Ethiopia and Sudan contributed to the determination of the genetic risk factors of BC.

In Sudan, two studies were performed, the first one reported 33 BRCA1 point mutations, found in 59 Central Sudanese premenopausal BC patients. [43]. The second study characterized germline BRCA1/2 mutations in patients (34 females, one male) selected by diagnosis within age 40 years or male gender. A total of 33/35 patient were found to carry 60 BRCA1/2 variants, among them, 17 were novel, 22 reported in populations from various geographic areas and 21 reported worldwide. The most frequent mutations found are in BRCA1 (c.3999delT, c.4065_4068delTCAA, c.557C>A, c.2458A>G, c.5090G>A) and in BRCA2 (c.3195_3198delTAAT, c.6406_6407delTT, c.8642_8643insTTTT, c.6101G>A, c.68-7delT) [44].

The most relevant studies in Ethiopia targeted the implication of the *HER* gene. This Proto-oncogene plays an important role in the carcinogenesis and the prognosis of BC [21]. Many studies have been conducted to explore the association between the *HER2* Ile655Val polymorphism and BC risk, a significant association among Africans was found for Val/Val *v.* Ile/Ile genotypes: odds ratio = 78, 95% confidence interval = 1.94–39.72, *p* = 0.35 for heterogeneity; for the recessive model Val/Val *v.* Ile/Val + Ile/Ile: odds ratio = 8.60, 95% confidence interval = 1.92–38.48, *p* = 0.31 for heterogeneity [21].

### Central Africa region

In this region, only Democratic Republic of Congo have studied the *BRCA1/2* genes in a family with a severe history of BC at young ages. This genetic analysis revealed the presence of the c.2389_2390delGA mutation at the heterozygous state in all BC family members, the mutation leads to a frameshift at codon 797 of the *BRCA1* gene (p.Glu797fs) [45].

A summary of the extracted data from our literature review, the panel of genes illustrated in Table 1 represents the genes associated with BC in the different African populations.

**Table 1.** Panel of genes associated with BC in African populations

	Northern				Southern	Western		Eastern	Central
Country	Algeria	Tunisia	Egypt	Morocco	South Africa	Nigeria	Senegal	Sudan	Democratic Republic of the Congo
Genes	BRCA1 BRCA2	BRCA1 BRCA2 HER2 ESR1 P53	BRCA1 BRCA2 P53 PON1 XPD	BRCA1 BRCA2 APOBEC3A APOBEC3B ARID1B ATR MAP3K1 MLL2 MLL3 NCOR1 RUNX1 SF3B1 SMAD4 TBX3 TTN MTHFR HER2	BRCA1 BRCA2 CHEK2 PALB2 RAD50 MTHFR hMLH1	BRCA1 BRCA2 LEPR	BRCA1	BRCA1 BRCA2	BRCA1 BRCA2

## Discussion

BC is the most commonly diagnosed cancer in African women and the main cause of death from cancer diseases. The evaluation of the BC genetic risk factor provided support to understand the relationship between genomics and cancer. Only nine of 54 countries in Africa have studied few genes involved in BC. In this review, data showed 27 distinct genes in Africa (Table 1). Mutations in *BRCA1* and *BRCA2* genes were identified in all five regions of the continent.

According to our literature review, the BRCA1 c.798_799delTT mutation was found recurrent in the three North African countries, Algeria [11] Tunisia [14] and Morocco [31] but absent in other African regions. Moreover, the mutation c.181T>G on BRCA1 was commonly reported only in the Algerian [11] and Moroccan [31] populations. Another BRCA1 mutation c.2612C>T was identified in Algeria [11] and Tunisia [17]. The BRCA2 mutations c.-26G>A, c.7242A>G and c.8503T>C were identified in two NA countries, Algeria [11] and Tunisia [14].

Furthermore, many common mutations in *BRCA1/2* were found in other regions, especially in Western Africa, where three common mutations with *BRCA1* (c.2082C>T, c.2311T>C, c.3548A>G) and two common *BRCA2* mutations (c.3396A>G, c.3807T>C) were identified [40]. These inter-regions similarities were also observed between South Africa and two NA countries: the c.1504_1508del BRCA1 mutation is common between South Africa [46] and Tunisia [17], and the c.185delAG BRCA1 between South African [37] and Egyptian [24] populations. More details concerning mutations retrieved in African countries are listed in Table 2.

**Table 2.** Mutation detection methods used in African Studies

	Northern				Southern	Western		Eastern	Central
Country	Morocco	Algeria	Tunisia	Egypt	South Africa	Nigeria	Senegal	Sudan	Democratic Republic of the Congo
BRCA1	c.68_69delAG c.181 T > G c.798_799delTT c.3279delC c.1016dupA c.4942A>T c.5062-5064delGTT c.5095C>T	c.2082C>T c.2311 T > C c.2612C>T c.3418A>G c.3548A>G c.4308 T > C c.181 T > G c.798_799delTT	c.798_799delTT c.1504_1508delTTAA c.211dupA c.2612C>T c.2311 T > C c.3022A>G c.4308 T > C	c.185delAG c.624C>T	c.185delAG c.448insA c.1127insA S451X c.4957insC c.1504_1508del	IVS2 + 1G>A c.252delAA c.2311delAAGAA c.2082C>T c.2311 T > C c.3548A>G c.5123C>T	c.1949_1950delTA	c.3999delT c.4065_4068delTCAA c.557C>A c.2458A>G c.1846_1848delTCT c.1088A>G	c.2389_2390delGA
BRCA2	c.517-1G>A c.3381delT c.5073dupA c.7110delA c.7235insG	c.-26G>A c.865A>C c.3396A>G c.3807 T > C c.7242A>G c.7397C>T c.8503 T > C c.1310_1313delAAGA	c.1313dupT c.7654dupT c.7242A>G c.-26G>A c.681 + 56C>T c.8503 T > C c.1310_1313del	c.999delTCAAA c.2256 T > C c.8934G>A	c.5771_5774del c.6447_6448dup c.7934delG c.582G > A	c.1222delA c.1538delAAGA c.1590delA c.681 + 56C>T c.3396A>G c.3807 T > C c.8755–66 T > C	c.296-7dupT c.5710C>G	c.3195_3198delTAAT c.6406_6407delTT c.8642_8643insTTTT c.6101G>A c.68-7delT	
Mutation detection methods	PCR, bi-directional sequencing, targeted direct sequencing.	PCR, PCR-SSCP, High-Resolution Melting (HRM) curve analysis, direct sequencing	PCR, direct sequencing.	PCR, single-strand conformational polymorphism (SSCP), Heteroduplex analysis (HDA), Cloning Vector, DNA Sequencing.	PTT and SSCP/HA analysis, PCR, Manual sequencing, Sanger sequencing, MLPA, Next-Generation Sequencing (NGS), Whole Exome Sequencing.	PCR, direct sequencing.	PCR, direct sequencing.	PCR, Protein truncation test (PTT), denaturing high performance liquid chromatography (DHPLC), direct sequencing.	
Reference	[29–31]	[11]	[14, 17, 18]	[24]	[37, 38, 46]	[40]	[42]	[43, 44]	[45]

Our literature review revealed that the majority of genes were studied in the NA (18 genes) . Whereas, only six genes were studied in SA. We performed a functional analysis using the online tool ToppGene (https://toppgene.cchmc.org) to explore the biological function and pathways of the studied genes in both of NA (Table 3) and SA (Table 4). The functional analysis revealed a strong similarity between the candidate genes from NA and SA regions. Furthermore, the biological pathways ‘Role of BRCA1, BRCA2 and ATR in Cancer Susceptibility’ and ‘Breast Cancer’ were found in the top enriched terms for NA and SA regions with a *p* of 1.074×10^−8^ (2.09×10^−10^) and 2.804×10^−5^ (2.69×10^−3^), respectively.

**Table 3.** Top enriched terms and biological pathways identified by functional analysis of breast cancer candidate genes studied in Northern Africa

Enrichment term	ID	Source	*p* value
Enriched pathways			
Role of BRCA1, BRCA2 and ATR in cancer susceptibility	M9703	MSigDB C2 BIOCARTA (v6.0)	1.074 × 10^−8^
Cell Cycle: G2/M checkpoint	M8560	MSigDB C2 BIOCARTA (v6.0)	3.467 × 10^−6^
Cell Cycle: G1/S check Point	M648	MSigDB C2 BIOCARTA (v6.0)	5.595 × 10^−6^
Breast cancer	1435207	BioSystems: KEGG	2.804 × 10^−5^
Fanconi anemia pathway	377262	BioSystems: KEGG	4.386 × 10^−5^
Pancreatic cancer	83108	BioSystems: KEGG	6.917 × 10^−5^
Chronic myeloid leukemia	83116	BioSystems: KEGG	9.437 × 10^−5^
Enriched gene ontology term			
Chromosome breakage	GO:0031052	GO	1.039 × 10^−8^
Programmed DNA elimination	GO:0031049	GO	1.039 × 10^−8^
Female sex differentiation	GO:0046660	GO	1.127 × 10^−7^
Chromosome organization	GO:0051276	GO	1.3 × 10^−7^
Positive regulation of chromosome organization	GO:2001252	GO	1.568 × 10^−7^
Positive regulation of macromolecule biosynthetic process	GO:0010557	GO	2.852 × 10^−7^
DNA damage response, signal transduction by p53 class mediator resulting in transcription of p21 class mediator	GO:0006978	GO	2.894 × 10^−7^

**Table 4.** Top enriched terms and biological pathways identified by functional analysis of breast cancer candidate genes studied in southern Africa

Enrichment term	ID	Source	*p* value
Enriched pathways			
Role of BRCA1, BRCA2 and ATR in Cancer Susceptibility	M9703	MSigDB C2 BIOCARTA (v6.0)	2.09 × 10^−10^
ATM Signaling Pathway	M10628	MSigDB C2 BIOCARTA (v6.0)	1.24 × 10^−7^
Homologous recombination	83046	BioSystems: KEGG	1.15 × 10^−6^
Fanconi anemia pathway	377262	BioSystems: KEGG	2.82 × 10^−6^
Cell Cycle: G2/M Checkpoint	M8560	MSigDB C2 BIOCARTA (v6.0)	7.44 × 10^−5^
Platinum drug resistance	1404797	BioSystems: KEGG	6.99 × 10^−4^
Breast cancer	1435207	BioSystems: KEGG	2.69 × 10^−3^
Enriched gene ontology term			
Double-strand break repair	GO:0006302	GO	2.53 × 10^−9^
Intrinsic apoptotic signaling pathway in response to DNA damage	GO:0008630	GO	4.28 × 10^−8^
Negative regulation of DNA metabolic process	GO:0051053	GO	4.76 × 10^−8^
Regulation of DNA metabolic process	GO:0051052	GO	5.96 × 10^−8^
Strand displacement	GO:0000732	GO	8.42 × 10^−8^
Meiotic metaphase I plate congression	GO:0043060	GO	1.21 × 10^−7^
DNA repair	GO:0006281	GO	3.21 × 10^−7^

GO, Gene Ontology; KEGG, Kyoto Encyclopedia of Genes and Genomes; MSigDB, The Molecular Signatures Database.

In the remaining regions, a modest number of genes have been studied only in few countries (Nigeria, Senegal, Sudan, etc.), this may reflect the lack of clinical monitoring for BC patients and control of the disease.

The screening for different types of gene mutations to determine their origin, either somatic or germinal, or either founder or recurrent mutations helped to characterize the population specificities toward genes and mutations found in populations across the continent. Researchers in American continent have studied the genetic factors of BC through decades using different types of technologies, from RFLP-PCR to Whole Exome Sequencing, these studies have largely contributed to define major and minor genes and mutation incidence. Several mutations, mainly in *BRCA1/2*, have been described in both American and African populations, such 185delAG [46], 1742insG, 2630del11, 4241delTG, 9045delGAAA and delEx12 [47]. A wide spectrum of mutations in other genes associated with BC have been identified but have not been significantly reported in both continents [48, 49]. Many mutations described in the American studies have not been described or are rarely found in the African continent, such as *VS511G.A, S955X, R1443X, E1644X, 943ins10, C64Y, M1775R, Q1090X* and *Y101X* [49]. A common phylogenetic relation between American and African populations would explain the recurring mutations found among them, and the genetic history between women with BC found in North or South America. The diversity of ethnicity among the American populations has an important role in explaining these findings. Indeed, a significant number of women diagnosed with BC in North America have African or Latino-Hispanic origins and a relevant number of mutations found in these women have been described in African women with BC [49]. However, many detected mutations in the American women with African-related origins have not been described in African studies. Considering these undescribed mutations in African BC women, it would be relevant to complete the African genetic risk factor panel in forthcoming BC genetic studies.

Our literature search revealed that only nine African countries had studied the genes involved in BC. Unlike, in Europe, where the studies covered 15 countries, which gives a valuable information regarding BC genetics in Europe. Indeed, different studies have been carried out through the European continent to determine the role and the implication of *BRCA1* and *BRCA2* genes in BC, as well as detect novel mutations [50–72]. Other genes have been studied in both African and European continents and revealed to be associated with BC, such as *CHEK2* [52, 65, 73–75], *TP53* [73, 76, 77], *MTHFR* [78], *PALB2* [79–81]. Whereas, other genes have been proved involved in BC development solely in Africa such as *APOBEC3A, APOBEC3B, ARID1B, NCOR1, SMAD4, MAP3K1, HER2, HER* and *RAD50* or only in Europe such as *RAD51* [82–84], *COMT* [85, 86], *CYP17* [86], *MRE11A* [61, 87], *CYP19* [87] and *MDM2* [88].

The ethnic similarity between the different regions of Europe and Africa can guide African scientists to well investigate the genes involved in BC. For instance, research in North Africa should explore other genes, such as *GSTM1*, *GSTT1* found in Portugal [89] and *PALB2*, *ATM*, *TNFRSF11A* found in Spain [80, 90, 91].

The investigation in Asia were more important. Indeed, many studies exploring the major and minor genes involved in BC were performed in different Asian countries. Several Meta-analyses and reviews were also conducted [21, 92, 93].

For *BRCA1* and *BRCA2* genes, several germline mutations were identified and listed in the National Comprehensive Cancer Network (NCCN) guidelines, however, no data were reported concerning African studies [94]. The genetic aetiology of hereditary BC has not yet been fully elucidated. Although germline mutations of high-penetrance genes such as *BRCA1/2* were shown implicated in development of BC hereditary, at least half of all BC families are not linked to these genes [95]. For example, in China 42 deleterious germline mutations were identified in 21 genes, including 18.2% BRCA1 or BRCA2 mutations, 3% TP53 mutations, 5.1% DNA mismatch repair gene mutations, 1% CDH1 mutations, 6.1% Fanconi anemia pathway gene mutations, and 9.1% mutations in other genes [95].

*CYP*, *CHEK2* and *MTHFR* were also studied and their involvement in BC showed that they may modify susceptibility to BC [70, 96, 97]. *TNF-alpha, Enos*, *RAD50, CCND1, NBS1* and *SULT1A1* genes were studied in Asia and the results exclude the possible role of *RAD50* and *NBS1* in familial BC [59]. *SULT1A1* may be a low-penetrant risk factor for developing BC in the Asian population [66]. Although the clinical significance of newly identified low-penetration genetic mutations has not yet been fully appreciated in Asia, these new findings provide valuable epidemiological information for future studies of BC in the Asian population.

Unlike Africa, many studies in Asia have used recent techniques such as NGS and Whole Exome Sequencing that have contributed to the development of genetic screening panels [55, 98, 99]. For example, a test panel has been developed in accordance with NCCN, which has proposed guidelines for the genetic testing of the *BRCA1* and *BRCA2* genes, based on studies in western populations. However, like in Africa, there is still a gap in the availability of genetic counselling and genetic testing in Asian countries due to financial facilities, access and inaccurate reporting of a family history of cancer.

The variety of options now accessible for the patient and physician in making appropriate and timely decisions in hereditary breast and ovarian cancer has triggered a daily increase in the demand for mutation analysis of the *BRCA1/2* genes on the other continents. Therefore, it is necessary for African scientists to implement these testing and analysis, to better characterize BC genetic risk factors.

The analysis of the African genetic studies reported in our literature search revealed that the screening of genes and mutations related to BC in African countries is less illustrative compared with other continents. Therefore, it is necessary to encourage researchers in Africa to characterize more genes involved in BC, to better target the diagnosis and guide specific and efficient therapeutic strategies for African community.

The insufficiency of reliable data on BC in Africa and the increase of mortality prevalence and incidence rates could be linked with precarious socioeconomic criteria. For instance, the weakness of the health system, the lack of health insurance and coverage, the limited access to medication, the scarcity of care facilities and counselling, the deficiency of genetic testing and low income. In addition, the lack of support for scientific research in several African countries also contributed to the spread of the disease.
